# One potential hotspot *SLC25A20* gene variants in Chinese patients with carnitine-acylcarnitine translocase deficiency

**DOI:** 10.3389/fped.2022.1029004

**Published:** 2022-11-07

**Authors:** Xiaoli Li, Jian Shen

**Affiliations:** Department of Pediatrics, Affiliated Hangzhou First People’s Hospital, Zhejiang University School of Medicine, Hangzhou, China

**Keywords:** CACT deficiency, *SLC25A20* gene, c.199–10T > G, hotspot mutation, single nucleotide polymorphism detection

## Abstract

**Background:**

Carnitine-acylcarnitine translocase deficiency (CACT deficiency) is a rare and life-threatening autosomal recessive disorder of mitochondrial fatty acid oxidation caused by variant of *SLC25A20* gene. The most prevalent missense variant in the *SLC25A20* gene in Asia was c.199–10T > G. Due to the c.199–10T > G variant, CACT deficiency is a severe phenotype.

**Materials and Methods:**

Herein, we present a neonatal case with c.199–10T > G variant in China and analyze the clinical, biochemical, and genetic aspects of 78 patients previously identified with CACT deficiency.

**Results:**

The patient presented with a series of severe metabolic crises that rapidly deteriorated and eventually died 3 days after delivery. The sequencing of the patient's genome indicated that he was homozygous for the c.199–10T > G variant. 30 patients were found to have the c.199–10T > G mutation, of which 23 were Chinese and 22 were afflicted by the c.199–10T > G splicing variation. In China, c.199–10T > G allele frequency was 82.6%.

**Conclusion:**

In CACT deficiency, prompt recognition and treatment are critical. Our data suggested that c.199–10T > G may be a potential hotspot *SLC25A20* gene mutation in the Chinese population. Detection of single nucleotide polymorphism is possible for high-risk patients and parents in China.

## Introduction

Carnitine-acylcarnitine translocase deficiency (CACT deficiency, OMIM # 212138) was first described by Stanley CA et al. in 1992 (1). It is a rare and life-threatening autosomal recessive disorder with an incidence of 1:60,000 in Hongkong and 1:1,017,593 in Zhejiang province China (2, 3). CACT deficiency, encoded by the *SLC25A20* gene on chromosome 3p21.31, is the cause of this condition (4). To shuttle long-chain fatty acids through the inner mitochondrial membrane and into the mitochondrial matrix, where mitochondrial β-oxidation takes place, CACT is an essential part of the carnitine cycle (5). Mitochondrial β-oxidation serves as the primary energy source for cardiac and skeletal muscles, while ketogenesis in the liver fuels brains tissue during prolonged fasting and exercise (1, 6, 7). CACT deficiency is characterized by a wide spectrum of clinical manifestations including hypoketotic hypoglycemia, hyperammonemia, liver dysfunction, cardiomyopathy, severe neurologic impairment and progressive myopathy (8).

There are nine exons in the *SLC25A20* gene, which produces a 301-amino-acid protein (9). At least 42 pathogenic variants have been detected in mutation databases like the HGMD around the world (Human Gene Mutation Database, www.hgmd.org). There are 20 missenses, 10 small deletions, 2 small insertions, 1 small indel, 4 large deletions, and 5 splicing mutations in the mutation spectrum (10). *SLC25A20* gene missense variation c.199–10T > G was the most frequent in Asia. In our study, approximately 37.5% of pathogenic variants fall within this umbrella.

Though the spectrum of CACT deficiency is wide and continuous, there are two distinct clinical subtypes: a neonatal-onset severe form and an infancy-onset milder form (11). Severe classic presentation occurs at birth and has an extremely poor prognosis, with severe illness and debilitating symptoms. Moderate myopathy and hepatomegaly are seen in milder cases with more accessible residual transporter protein. Metabolic decompensation can be prevented and the prognosis improved with early detection and medicinal intervention (12).

In the present study, we described a patient with CACT deficiency which was failed to diagnosed and treated promptly and then leaded to rapid illness progression and eventual death. Furthermore, Patients previously diagnosed with CACT deficiency was reviewed systematically by describing the clinical, biochemical, and genetic characteristics and treatment to improve our understanding of this rare disorder.

## Materials and methods

### Case report

**Figure 1:**
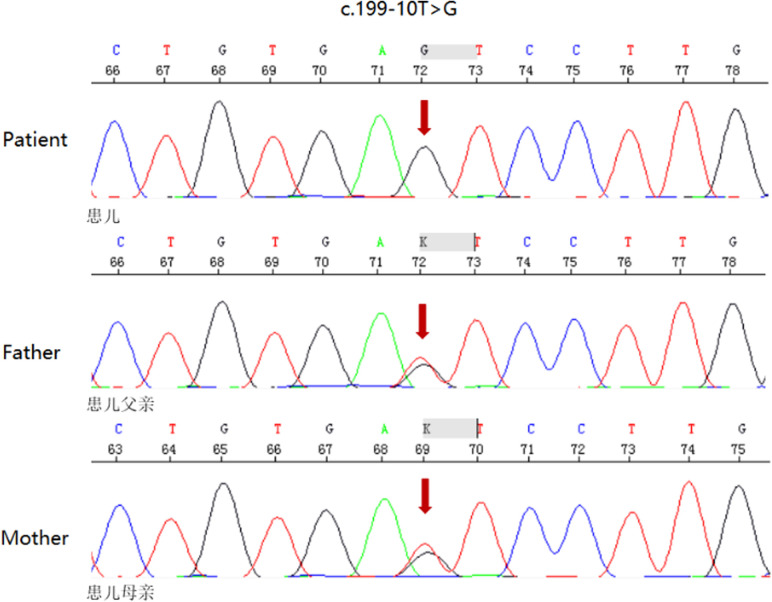
DNA sequence of SLC25A20 gene (a homozygous mutation of patient for maternally and paternally-inherited c.199–10 T > G variant. Parents were heterozygous carriers of the mutation. Mutation sites are indicated by red arrows)

The patient was born at full term through a cesarean section. At 1 and 5 min, the baby's Apgar score was 10. The parents were healthy and had no history of consanguineous marriage. The mother's first child died at two days old from asphyxia, arrhythmia, and cardiac arrest. The baby seemed fine until he was 28 h old when he became very sleepy and showed no interest in breastfeeding. The patient was taken to the neonatal intensive care unit (NICU) for a checkup. During a physical exam, the baby's body temperature was 34.6°C, his blood pressure was 72/44 mmHg, and his SpO2 was 95%. Table 1 gives detailed information about the patient's health. After a test for sepsis was done, he was given antibiotics, and a 6.5 mg/ka/minute glucose infusion was started. The patient had recurrent ventricular tachycardia, bradycardia, and complete right bundle branch block between the ages of 47 and 51 h. Lidocaine, epinephrine, and milrinone were used to treat the patient for several days after the attack. Several attempts to save her life failed, and she died 3 days after giving birth. At 48 h, a small spot of dried blood was taken as a sample. Acylcarnitine profile by MS/MS analysis showed C14-acylcanitine, 1.13 µM (0.07–0.4); C16-acylcanitine, 14.37 µM (0.49–6); C16:1-acylcanitine, 1.40 µM (0.02–0.49); C18-acylcanitine, 3.73 µM (0.24–1.90); C18:1-acylcanitine, 5.83 µM (0.38–2.92), which were identified to have CPT2 (carnitine palmitoyl-transferases 2) deficiency or CACT deficiency. The patient's genomic DNA was taken out. High-throughput sequencing found that the patient's *SLC25A20* gene had a homozygous c.199–10T > G splice site change (Figure 1). The child was diagnosed with CACT deficiency based on the signs and symptoms. Both parents were heterozygous carriers of the variation, but neither had any obvious symptoms.

**Table 1:** Summary of the patient with CACT deficiency biochemistry, ECG and Echo

Age at detection	28 h (at admission)	47 h	51 h
ALT (U/L)	20	26	–
AST (U/L)	74	74	87
HLD (U/L)	461	592	784
Lactate (mmol/L)	6.0	7.2	–
Ammonia (µmol/L)	128	355	433
CK (U/L)	384	418	596
CKMB (U/L)	66	136	168
Ca^++^ (mmol/L)	2.2	1.49	1.83
K ^+ ^ (mmol/L)	5.98	6.52	6.59
TNI (µg/L)	–	0.03	–
Glucose (mmol/L)	1.9	1.49	1.3
ECG	–	VT/CRBBB/II-AVB	Bradycardia/VF/Unusually broad and changing QRS wave complex
Echo	Left ventricular wall motion incoordination (EF55%)	–	Left ventricular wall motion incoordination (EF30%)

Cutoff value: ALT(alanine transaminase):9–50 U/L; AST(aspartate transaminase):15–40 U/L; HLD(lactate dehydrogenase):97–350 U/L; Lactate:1.5–2.5 mmol/L; Ammonia:18–72 µmol/L; CK(creatine kinase): 24–195 U/L; CK-MB(creatine kinase-MB):0–24 U/L; Ca^++^2.0–2.6 mmol/L; K ^+ ^3.5–5.3 mmol/L; TNI(Troponin I) < 0.01 µg/L; VT, ventricular tachycardias; II-AVB, second-degree atrioventricular blocks.

### Literature search

All patients previously genetically diagnosed with CACT deficiency were reviewed in the study. The literature search for “Carnitine-acylcarnitine translocase deficiency,” “Carnitine-acylcarnitine translocase,” “*SLC25A20* gene,” “CACTD,” as keywords on PubMed, Elsevier and Medline from 1992 to June 2022. (Table 2).

**Table 2:** Presenting features of patients with CDCT deficiency (1, 3, 5, 8–10, 13–32).

Patient ID	Ethnicity	Age at onset	Current age/age of death	Hypoglycemia	Hyperammonemia	CK	ALT	Arrhythmias	Cardiac hypertrophy	Cardiac arrest	Hepatomegaly	Seizures	Poor response/Hypotonia	dyspnea	Other futures	SLC25A20 genotype
1	Malaysia	24 h	2.5 months	1	1	NA	NA	NA	NA	NA	NA	1	NA	NA	(C16 + C18:1)/C2↑	Homozygous c.199–10T > G
2	Malaysia	24 h	5 day	1	1	NA	↑	AVB/VT/VF/bradycardia	NA	NA	NA	NA	1	NA	(C16 + C18:1)/C2↑	Homozygous c.199–10T > G
3	Chinese	24 h	3 year	1	1	↑	NA	RBBB/AVB	LV hypertrophy	1	NA	1	NA	NA	NA	Homozygous c.199–10T > G
4	Vietnamese	28 h	6 months	1	1	↑	NA	Broad complex VT	NA	NA	NA	NA	NA	NA	NA	Homozygous c.199–10T > G
5	Chinese	14.5 h	4 years lived	1	1	↑	NA	Bradycardia	cadiomyopathy with thickened LV/RV and septum	1	NA	1	NA	NA	C16, C18↑	Homozygous c.199–10T > G
6	Chinese	8 days	14 months lived	1	1	↑	↑	Bradycardia	Hypertrophic cardiomyopathy	NA	NA	NA	NA	1	Rhabdomyolysis C16, C18↑	Homozygous c.199–10T > G
7	Vietnamese	12 h	5 months lived	1	1	↑	NA	VT	NA	1	NA	1	NA	NA	C16, C18↑	Homozygous c.199–10T > G
8	Chinese	25 min	71 h	1	1	↑	NA	NA	NA	1	NA	1	NA	NA	(C16 + C18:1)/C2↑	Homozygous c.199–10T > G
9	Chinese	24 h	48 h	1	1	↑	NA	AVB/VT	NA	NA	NA	1	1	1	C16, C18↑	Homozygous c.199–10T > G
10	Thailand	10 h	2 years and 8 months	1	1	↑	↑	NA	Hypertrophic cardiomyopathy	1	1	NA	NA	1	C16, C18↑	Homozygous c.199–10T > G
11	Thailand	48 h	4 months	1	1	↑	↑	NA	Hypertrophic cardiomyopathy	1	1	NA	1	NA	C16, C18↑	Homozygous c.199–10T > G
12	Chinese	Early Neonatal	2 months	1	NA	↑	NA	VT	Cardiac hypertrophy	1	NA	NA	1	1	C16, C18↑	Homozygous c.199–10T > G
13	Chinese	1 day	3 days	NA	NA	NA	NA	1	Cardiac hypertrophy	1	NA	NA	1	NA	C16, C18↑	Homozygous c.199–10T > G
14	Chinese	Treated from birth	19 months lived	NA	NA	NA	NA	VT	NA	NA	NA	NA	NA	NA	C16, C18↑	Homozygous c.199–10T > G
15	Chinese	2 days	2 days	1	1	NA	NA	1	Cardiac hypertrophy	1	NA	NA	NA	NA	C16, C18↑	Homozygous c.199–10T > G
16	Chinese	41 h	41 h	NA	NA	NA	NA	NA	NA	1	NA	NA	NA	NA	NA	Homozygous c.199–10T > G
17	Chinese	32 h	32 months	1	1	NA	NA	NA	Hypertrophic cardiomyopathy	NA	NA	NA	NA	NA	NA	Homozygous c.199–10T > G
18	Chinese	28 h	38 h	NA	NA	NA	NA	VT/Bradycardia	NA	1	NA	NA	NA	NA	NA	Homozygous c.199–10T > G
19	Chinese	61 days	62 days	1	NA	↑	↑	NA	NA	NA	NA	NA	1	NA	NA	Homozygous c.199–10T > G
20	Chinese	In 7 days	In 7 days	1	1	NA	NA	NA	Hypertrophic cardiomyopathy	1	1	NA	NA	NA	C14↑, C16↑, C18↑, C16:1↑, C18:1↑	Homozygous c.199–10T > G
21	Chinese	In 7 days	In 7 days	1	1	NA	NA	NA	Hypertrophic cardiomyopathy	NA	1	NA	NA	NA	C14↑, C16↑, C18↑, C16:1↑, C18:1↑	Homozygous c.199–10T > G
22	–	1 day	4 years lived	1	1	NA	NA	symptomatic tachyarrhythmia	cardiac hypertrophy	NA	NA	NA	NA	NA	C16↑, C18↑	Homozygous c.199–10T > G
23	Chinese	2 days	3 days	1	1	↑	↑	VT/IRBBB Bradycardia	NA	1	NA	NA	1	NA	C14↑, C16↑, C18↑, C16:1↑, C18:1↑	Homozygous c.199–10T > G
24	Chinese	24 h	9 years lived	1	1	↑	↑	Broad complex tachyarrhythmia atrial flutter	biventricular hypertrophy	NA	NA	1	NA	NA	NA	c.109C > T c.199–10T > G
25	Chinese	24 h	71 h	1	1	↑	↑	1	NA	NA	NA	1	1	NA	C16↑, C18:1↑	c.199–10T > G c.1A > G
26	Chinese	24 h	6 days	1	1	↑	↑	NA	NA	NA	NA	NA	1	NA	C16, C18, (C16 + C18:1)/C2↑	c.199–10T > G c.1A > G
27	Chinese	–	1 year and 3 months	1	1	↑	NA	NA	NA	NA	NA	NA	NA	NA	C14↑, C16↑, C18↑, C16:1↑, C18:1↑	c.199–10T > G c.1A > G
28	Japanese	48 h	2 years and 9 months	1	NA	NA	↑	NA	NA	NA	NA	NA	1	1	C14, C16, C18↑	c.199–10T > G c.576G > A
29	Chinese	2 days	3 days	1	1	↑	↑	1	cardiac hypertrophy	1	NA	NA	1	1	C16↑, C18↑	c.199–10T > G c.719-8_c.719-1dupCCCCACAG
30	Chinese	4 days	8 days	1	1	↑	↑	1	NA	NA	NA	1	1	1	C16↑, C18↑	c.199–10T > G c.719-8_c.719-1dupCCCCACAG
31	Pakistani descent	1 month	lived	NA	NA	NA	NA	NA	NA	NA	NA	NA	NA	NA	NA	Homozygous c.82G > T
32	Pakistani descent	2 months	lived	1	NA	NA	NA	NA	LVH, Impaired contractility	NA	NA	NA	NA	NA	C16↑	Homozygous c.82G > T
33	Pakistani descent	–	lived	NA	NA	NA	NA	NA	NA	NA	NA	NA	NA	NA	NA	Homozygous c.82G > T
34	Pakistani descent	9 days	lived	1	NA	NA	NA	NA	NA	NA	NA	NA	NA	NA	C16↑	Homozygous c.82G > T
35	Pakistani descent	–	lived	NA	NA	NA	NA	NA	NA	NA	NA	NA	NA	NA	NA	Homozygous c.82G > T
36	Pakistani descent	1 month	lived	NA	NA	NA	NA	NA	NA	NA	NA	NA	NA	NA	NA	Homozygous c.82G > T
37	Pakistani descent	4 months	lived	1	NA	NA	NA	NA	Mild left ventricular hypertrabeculation	NA	NA	NA	NA	NA	NA	Homozygous c.82G > T
38	Malaysia	33 h	33 h	1	NA	NA	NA	NA	NA	1	1	NA	NA	NA	NA	c.109C > T c.706C > T
39	Indian	12 h	7 days	1	NA	NA	NA	Bradycardic arrest (rosc after 10 min)	NA	1	NA	1	NA	NA	NA	c.82G > T c.706C > T
40	Caucasian	10 h	3 days	1	1	NA	NA	VT	NA	1	NA	NA	NA	NA	NA	Homozygous c.646G > T
41	Iranian	24 h	11 years lived	1	1	↑	NA	SVT	NA	1	NA	1	NA	NA	NA	Homozygous c.67G > T
42	Guyana	15 h	3 years lived	1	1	NA	↑	NA	NA	NA	NA	1	NA	NA	NA	Homozygous c.110G > C
43	Caucasian	2 days	10 years lived	1	1	↑	NA	NA	NA	NA	NA	1	NA	NA	NA	c.50G > C c.326 + 1 delG
44	Iranian	Treated from birth	19 months lived	NA	1	NA	NA	AVT/VT	NA	NA	NA	NA	NA	NA	NA	Homozygous c.417 + 1G > A
45	Irish	32 h	16 months lived	1	1	↑	NA	AV	NA	1	NA	NA	NA	NA	Mild coagulation disorder	c.326 + 1delG (Splice donor); c.691G > C
46	New Zealanders	36 h	12 months lived	1	1	↑	NA	NA	echogenic myocadium	NA	NA	NA	NA	NA	NA	c.804delG
47	Iranian	5 months	–	1	NA	NA	NA	NA	Hypertrophic cardiomyopathy	NA	NA	NA	NA	NA	NA	Homozygous c.67G > T
48	Caucasian	2 months	–	1	NA	NA	↑	NA	NA	NA	NA	NA	NA	NA	Hypokalemia	c.397C > T deletion c.779_781delAAG
49	Caucasian	20 days	–	1	1	NA	NA	NA	NA	NA	NA	NA	NA	NA	c.823C > T is nonsense mutation	large 26 kb deletion encompassing exons 5–9
50	Iranian	10 months	–	1	NA	↑	↑	NA	NA	NA	NA	1	NA	NA	NA	c.160_163del4ins4 and c.804delG
51	–	1 month	–	1	NA	NA	NA	1	NA	NA	NA	NA	NA	NA	NA	c.397C > T c.752_761del10
52	–	20 days	–	NA	NA	NA	NA	NA	NA	NA	NA	NA	NA	NA	NA	c.528delT c.496C > T
53	–	2 years	–	1	NA	NA	NA	NA	NA	NA	NA	NA	NA	NA	NA	c.168delT
54	Turkey	24 h	10 months	1	1	NA	↑	NA	NA	NA	NA	NA	NA	1	C16, C18, C18:1↑	Homozygous c.408C > A
55	Turkey	10 days	12 months	1	1	NA	↑	NA	NA	NA	NA	NA	1	1	C16↑	Homozygous c.270del
56	Turkey	24 h	52days	NA	1	NA	↑	NA	NA	NA	1	NA	NA	NA	C14, C16, C18, C18:1↑	Homozygous c.270del
57	Japanese	48 h	3 days	NA	NA	NA	NA	NA	NA	1	NA	NA	1	NA	C16, C18↑	c.576G > A c.106-2A > T
58	Dutch	Neonatal	9 years lived	1	NA	NA	NA	NA	NA	1	1	NA	NA	NA	NA	Insertion of a cytosine in bp 955–959
59	Dutch	36 h	24 months	1	NA	NA	NA	NA	Hypertrophic cardiomyopathy	NA	NA	NA	1	NA	NA	c.241G > A missense mutation
60	Dutch	24 h	12 years lived	1	NA	NA	↑	NA	Hypertrophic cardiomyopathy	1	1	NA	1	NA	NA	c.955insC mutation
61	Italian	1 month	6 months	1	1	NA	↑	1	NA	NA	NA	NA	1	1	C16, C18:1↑	c.718 + 1G > C c397C > T
62	Italian	2 days	8 months lived	1	NA	NA	NA	Bradycardia and tachycardia	Hypertrophic cardiomyopathy	NA	1	1	1	NA	C16, C18:1↑	c.843 + 4-843 + 50del
63	Spanish	NA	4.5years lived	NA	NA	NA	NA	NA	NA	NA	NA	NA	NA	NA	C16, C18:1↑	c.532C > T c.159dupT c163delA
64	Spanish	40 h	4 months	1	NA	↑	↑	NA	NA	NA	1	NA	NA	NA	NA	c.159dupT c163delA
65	Italian	72 h	3 years and 5 months lived	1	1	NA	↑	NA	Mild cardiomegaly	NA	NA	NA	1	NA	C16, C18:1↑	Homozygous c.842C > T
66	North American	18 h	2 years lived	1	1	↑	↑	NA	Hypertrophic cardiomyopathy	NA	NA	NA	1	NA	C12–C22↑	c.362delG c.691G > C
67	Australian	27 h	After 27 h	1	NA	NA	NA	NA	NA	NA	1	NA	NA	NA	C14, C16 and C6-dicarboxylic (adipyl) acylcarnitines↑	c.326delG c.609-3C > G
68	Arabs	32 h	9 days	1	1	↑	↑	1	NA	1	NA	NA	NA	1	C6,C14,C16↑	Homozygous c.609-3C > G
69	South Africans	12 h	8 months	1	1	NA	NA	NA	Biventricular hypertrophy	NA	NA	NA	NA	NA	C12,C16,C18↑	Homozygous c59G > A
70	Spanish	8 months	4 years lived	1	NA	↑	↑	NA	NA	NA	1	1	1	NA	C14, C18↑	c.160_163del4ins4c.536A > G
71	Moroccan	33 h	12 months	1	NA	NA	NA	NA	NA	NA	NA	NA	NA	NA	NA	Homozygous c.536A > G
72	Spanish	Treated from birth	16 years lived	NA	NA	NA	NA	NA	NA	NA	NA	NA	NA	NA	NA	
73	American	5 days	3 years	1	1	NA	NA	LBBB/Prolonged QT interval	Hypertrophic cardiomyopathy	NA	NA	NA	NA	NA	NA	c.84delT a 506-kb deletion
74	Chinese	–	2 months	1	1	NA	↑	NA	Hypertrophic cardiomyopathy	NA	1	NA	NA	NA	C12, C14, C16, C18, C16:1, C18:1↑	c.270delC c.804delG
75	Italian	18 h	2 years lived	1	1	↑	↑	Tachycardia and acute arrhythmias	Biventricular hypertrophy	NA	1	NA	NA	NA	C2↓, C16:0, C18:1, C18:2↑	Homozygous c.713A > G
76	Japanese	2 days	5 years lived	NA	1	NA	NA	Bradycardia	NA	NA	1	NA	NA	1	C16, C14:C3, C16 + C18:1/C2↑	Homozygous c.824G > A
77	Japanese	2 days	26 months	1	1	NA	NA	VT	NA	1	NA	1	NA	NA	C16, C14:C3, C16 + C18:1/C2↑	Homozygous c.824G > A
78	Japanese	30 min	5 years lived	1	NA	NA	NA	NA	Biventricular hypertrophy	NA	NA	NA	NA	1	C16, C14:C3, C16 + C18:1/C2↑	Homozygous c.824G > A
79	American	2 days	lived	1	1	↑	NA	NA	Left ventricular septal hypertrophy	NA	NA	1	1	NA	C16, C18:1/C2↑	c.397C > T c.658G > A

1, existence; ↑, increase; VT, ventricular tachycardia; VF, ventricular fibrillation; LBBB, left bundle branch block; RBBB, right bundle branch block; AVB, Atrioventricular block.

## Results

Individual case reports are available for all cases as online.

### Case for CACT deficiency

Over 30 years, 81 children with CACT deficiency were identified, 3 of which lacked genetic testing to determine the mutation sites. Therefore, in addition to the newly identified patient with CACT deficiency, we included 78 previously diagnosed patients in our sample.

### Genetic findings

Patients harbored either homozygous or heterozygous *SLC25A20* mutations. 42 different variations have been discovered. The most prevalent splicing variant was homozygous c.199–10T > G (23/79). Heterozygous c.199–10T > G splicing variation (7/79) and homozygous c.82G > T splicing variation (7/79) were quite prevalent variations. Each of the forty remaining variants was detected one to three times. The c.199–10T > G variant was identified in thirty patients (38.0%), of whom 73.3% (22/30) were Chinese. 29.1% (23/79) of the patients were Chinese, and 95.7% (22/23) were affected by the c.199–10T > G splicing mutation. The remaining sufferers were scattered in different nations. The frequency of the c.199–10T > G allele was 33.5% in all cases, whereas it was 82.6% in China.

### Biochemical and clinical specifications

Twenty of the thirty individuals with the c.199–10T > G variant exhibited clinical symptoms within 48 h (66.7%). There were arrhythmias in 18 patients (60%), cardiomyopathy in 13 patients (43.3%), hepatomegaly in 4 patients (13.3%), seizures in 9 patients (30%), hyperammonemia in 22 patients (73.3%), increase of CKMB and ALT in 17 patients (56.7%) and 12 patients (40%) respectively. 23 of 30 individuals (76.7%) perished due to the variation c.199–10T > G, which is linked to a severe phenotype.

7/79 cases were attenuated, and homozygosity for variation c.82G > T was confirmed. All of these patients of Pakistani heritage survived. Three patients were diagnosed with cardiomyopathy, although only one had seizures. There were no other clinical signs noted in these patients.

42/79 individuals harbored additional *SLC25A20* mutations. 25 out of 42 participants exhibited clinical signs within 48 h (59.6%). These patients exhibited associated clinical manifestations: 14/42 (33.3%) had arrhythmias, 15/42 (35.7%) had bouts of cardiomyopathy, 11/42(26.2%) had hepatomegaly, and 7/42(16.7%) had seizures. Ammonia, CK and ALT levels were elevated in 22/42(52.3%), 11/42(26.2%) and 14/42(33.3%) patients, respectively.

## Discussion

CACT deficiency appears to be very rare in the general population, except for a small number of ethnic subgroups. This study described the biochemical, clinical and genetic characteristics of patients with CACT deficiency, analyzed the distribution and ethnic specificity of the pathogenic genes, provided a theoretical basis of single nucleotide polymorphism detection, and thus contributed to the body of knowledge for early diagnosis and timely intervention in this rare disorder.

There is a wide variation in the prevalence of CACT deficiency among ethnic groups. About 30 cases of CTCT deficiency have been reported elsewhere, while there have been more than 50 reported in Asia. It is reported that the estimated incidence of CACT deficiency is 1/60,000 in Hongkong, 1/76,894 in Hunan, and at least 1/100,000 in Guangzhou China (2, 10, 15). Caucasian groups had a substantially lower incidence of CACT deficiency, which was reported to be 1:750,000–1:2,000,000. Indeed, the misleading clinical presentation, poor prognosis and the need to collect blood and urine specimens for metabolic investigation at an appropriate time in relation to the illness frequently limit the recognition of the disorder. For these reasons, the frequency of CACT deficient inborn defects is probably higher than recorded cases.

CACT deficiency is one of the most severe disorders of the carnitine transport system and mitochondrial fatty acid oxidation. The disorder results in deficient formation of energy-yielding substrates and toxicity of acylcarnitine accumulation which plays a pivotal role in the production of arrhythmias, and then presents a simultaneous dysfunction of the heart, liver, and skeletal muscle, associated with hypoketotic hypoglycemia (33). Severe classic manifestation, the most common, is accompanied by severe hypoketotic hypoglycemia, refractory hyperammonemia, elevated transaminase levels and CK, cardiomyopathy, and abrupt arrhythmias. The prognosis for those with the classic results is exceptionally dismal. Moderate myopathy and hepatomegaly are seen in milder cases, which are less common but do have more accessible residual transporter protein (11, 34, 35). Following an initial metabolic decompensation at birth, the neonate, in our case, developed hypoglycemia, hyperammonemia, and acute and severe arrhythmias before passing away. This may be related to the less residual enzyme activity and the increased accumulation of carnitine-acylcarnitine (33, 34). In our study, CACT deficiency due to the c.199–10T > G variation is a severe phenotype with a significantly higher mortality, arrhythmia, seizures, and hyperammonemia incidence than other variations, while CACT deficiency caused by the c.82G > T mutation is associated with milder phenotype (5). The most frequent mutation was a splicing site variation of c.199–10T > G.

Early recognition and timely treatment are crucial in CACT deficiency. NBS (Newborn screening) plays an important role in early detection of deficiency in enzymes of mitochondrial carnitine-acylcarnitinecycle. Most patients with CACT and CPT2 deficiency had a higher C12–C18:1 level than those without these (3). *SLC25A20* gene mutational analysis is required to identify CACT deficiency, but this can be done without CACT activity assessment. Once a CACT deficiency has been diagnosed, the proper treatment must be implemented. To begin, sufficient glucose must be provided to prevent lipolysis from being broken down. Fasting prevention with frequent meals, a diet rich in carbohydrate, restricting long-chain fatty acids, supplementing with medium chain triglycerides (MCT) and essential polyunsaturated fatty acids are recommended as long-term treatments for CACT deficiency. Administration of carnitine is controversial, on the one hand it exerts to prevent arrhythmias, and on the other hand it causes an increase of acylcarnitines, responsible of arrhythmias (26, 33, 35). Triheptanoin can ameliorate acute cardiomyopathy and increase survival in patients with severe long-chain fatty acid oxidation disorders (26). CACT deficiency can also be treated with skimmed breast milk (30).

There are nine exons in the *SLC25A20* gene, which spans more than 903 bp of genomic DNA on chromosome 3p21.31 (35). At least 42 pathogenic variants of *SLC25A20* have been discovered so far. In contrast to the majority of pathogenic variations, c.199–10T > G and c.82G > T were shown to be shared in Asian and Pakistani origins, respectively. The founder mutation c.82G > T was detected in people of Pakistani ancestry. Our study's Chinese patients were found to have a wide range of homology, with seven distinct variants. The most common variant, c.199–10T > G, suggests that c.199–10T > G may be a hotspot of *SLC25A20* gene mutation in the Chinese population. c.1A > G was detected only 3 times and was not yet found in other than Chinese populations, so it may be unique to the Chinese individuals. The remaining variant was detected only once or twice.

In conclusion, the biochemical, clinical, and genetic characteristics of Chinese patients with CACT deficiency identified in this investigation may aid early identification and intervention. In addition, it appeared from the data that the Chinese patients have a high degree of homozygosity. The c.199–10T > G variant, which is the most common one in this population, has the potential to be a hotspot *SLC25A20* gene mutation. As a result, economical and rapid single nucleotide polymorphism and the genotyping assay can be performed for high-risk patients and their parents in China. In addition, prenatal or presymptomatic diagnosis can be performed in siblings.

## Data Availability

All data, models, and code generated or used during the study appear in the submitted article.
